# Encouragement of American Medical Periodical Literature

**Published:** 1849-03

**Authors:** 


### EDITORIAL DEPARTMENT.

*Encouragement of American Medical Periodical Literature.* The disposition among the profession of this country to patronize foreign productions, rather than encourage those emanating at home, tends, in no small degree, to repress the growth of our national medical literature. The greater portion of medical works which compose the library of the American practitioner, are republished English productions. Next, in number, are translations of French and German works. Those of American origin occupy, relatively, but a small niche. Many persons seem to have an impression that a national currency, as regards medical literature, is out of the question, and that, to possess any special value, a book must come to us with the stamp of a foreign mint. The mere fact that a work has found a publisher abroad, seems to suffice for its successful reproduction with us; so that it is not an uncommon occurrence for a foreign author, who, with his own countrymen is scarcely known, to have considerable celebrity with us, in consequence of the much greater circulation which he acquires. In this respect, it is emphatically true, that, in the words of the poet, " distance lends enchantment to the scene." A puerile performance by some unfledged candidate for bibliographical reputation who happens to reside at London or Paris, in the minds of some, may take precedence of a work by one whose experience, acquirements, and integrity are sufficiently well known, but who, unfortunately for his claims upon his own countrymen, resides on this side of the Atlantic. We may perhaps express ourselves in rather too strong terms, but it must be apparent to all who have given the subject attention, that a disposition to overestimate what comes to us from abroad, and to underrate our domestic productions, is too common. This fact, without doubt, discourages many, who, otherwise, would aspire to the distinction of successful authorship, and, hence, tends to impoverish our national medical literature.

We shall not undertake to prove that this virtual concession of inferiority is wholly gratuitous. We leave this point to the reflections of our readers. It is one of the characteristics of the study of medical science, that it not only concerns phenomena which are essentially the same in all countries, but that every country may furnish abundant materials for investigation. The medical philosopher is not under the necessity of traversing oceans and territories to observe or collect objects for his pursuit, but they are always at hand. The human body is the same everywhere—its anatomy—its physiology—and (as a general remark) its pathology. To discover truth, and advance knowledge in this province of inquiry, cannot be a prerogative incident to place, but to industry and skill. Does the American character in the latter particulars fall below that of other countries? This is the question which, without discussion, we leave for our readers to answer. We will only say, that, unless the answer to this question be in the affirmative, it seems to us we are to look for the cause of the poverty of our native medical literature, in a great measure, to the source which we have indicated—viz: a disposition to encourage and patronize an imported literature to the exclusion of a proper respect for that which is of domestic origin. We by no means advocate that overweening attachment to indigenous literature which would deprive us of the benefits of foreign productions. We see the evils of excessive national pride, in this respect, in the difficulty which attends the transportation of scientific developments in France across the English Channel. The arrogant exclusiveness, and bitter prejudices of national pride, are to be avoided, not less than a deficiency of patriotism. The latter, in our country, at the present time, seems an evil to be corrected. So long as a considerable portion of the medical profession are bent on holding a kind of provincial dependency on a foreign country, by seeking with avidity republications and translations, (the only recommendation of many of which consist in the mere fact that they are republications and translations) neglecting, at the same time, the meritorious efforts of our brethren at home, just so long will our bibliographical medical literature continue impoverished, being made up, in a great measure, of notes and additions to foreign works, by those who are as competent to write books, as to occupy the more humble office of editor and annotator.

Much might be said on this subject, but we did not commence this article with any intention of offering even the few remarks which have been submitted. We designed to allude more particularly to *medical periodicals.* Medical Journals are issued in various parts of the United States, some of which certainly are conducted with ability, and posses great merit. Generally editorial labors are gratuitous, there being very few Journals, in this country, which afford any revenue over the expenses of publication. Yet, under these circumstances, many are with difficulty sustained, the patronage being inadequate even to the cost of publication. We have noticed, of late, announcements that several will be discontinued, unless better sustained, a pecuniary burthen for the issues falling upon the editor. Now, why is it, that an excellent journal, even of long standing, can hardly be sustained, with the gratuitous services of an able editor? One reason, doubtless, is, that the same spirit which determines preferences for foreign books, also tends to encourage foreign periodical literature, to the prejudice of the best efforts of American journalists. We shall content ourselves here with simply suggesting this as a topic of reflection for those who may honor these remarks with a perusal. Our thoughts were directed to it by a few editorial paragraphs contained in our esteemed contemporary, the Boston Medical and Surgical Journal, with which we will take the liberty of concluding this article:—

"Having been requested to notice the American reprint of the Lancet, by Messrs. Stringer & Townsend, of New York, we are justified in saying that the first number of a new volume looks extremely well. The work also contains, as every one knows, a great amount of useful matter. But instead of unqualifiedly recommending our medical brethren to patronise it, we prefer urging them to cherish our own periodicals, which need their fostering care. If American Journals of medicine are obliged to go begging, or be discontinued, there can be no native medical literature, and no advances will be made in respect to gathering in the experience of our surgeons and physicians. We advise, therefore, all American practitioners who prefer receiving a foreign periodical, as well as those who do not, to subscribe also for one of native growth, which will not cost so much, yet will have the pith of the European, with the medical intelligence, and medical papers and reports from the highest professional authorities of their own country. A love for home manufactures, home enterprise, and a desire to promote and enlarge the sphere of medical science in the Union, prompts to these observations."

